# Ketogenic diet improves behaviors in a maternal immune activation model of autism spectrum disorder

**DOI:** 10.1371/journal.pone.0171643

**Published:** 2017-02-06

**Authors:** David N. Ruskin, Michelle I. Murphy, Sierra L. Slade, Susan A. Masino

**Affiliations:** Department of Psychology, Neuroscience Program, Trinity College, Hartford, CT, United States of America; UPMC, FRANCE

## Abstract

Prenatal factors influence autism spectrum disorder (ASD) incidence in children and can increase ASD symptoms in offspring of animal models. These may include maternal immune activation (MIA) due to viral or bacterial infection during the first trimesters. Unfortunately, regardless of ASD etiology, existing drugs are poorly effective against core symptoms. For nearly a century a ketogenic diet (KD) has been used to treat seizures, and recent insights into mechanisms of ASD and a growing recognition that immune/inflammatory conditions exacerbate ASD risk has increased interest in KD as a treatment for ASD. Here we studied the effects of KD on core ASD symptoms in offspring exposed to MIA. To produce MIA, pregnant C57Bl/6 mice were injected with the viral mimic polyinosinic-polycytidylic acid; after weaning offspring were fed KD or control diet for three weeks. Consistent with an ASD phenotype of a higher incidence in males, control diet-fed MIA male offspring were not social and exhibited high levels of repetitive self-directed behaviors; female offspring were unaffected. However, KD feeding partially or completely reversed all MIA-induced behavioral abnormalities in males; it had no effect on behavior in females. KD-induced metabolic changes of reduced blood glucose and elevated blood ketones were quantified in offspring of both sexes. Prior work from our laboratory and others demonstrate KDs improve relevant behaviors in several ASD models, and here we demonstrate clear benefits of KD in the MIA model of ASD. Together these studies suggest a broad utility for metabolic therapy in improving core ASD symptoms, and support further research to develop and apply ketogenic and/or metabolic strategies in patients with ASD.

## Introduction

Autism spectrum disorder (ASD) is defined by poor sociability and communication alongside increased repetitive behaviors or limited behavioral repertoires. Although ASD is defined by specific behavioral criteria, there is a multitude of genetic and/or environmental contributors, and most often the etiology is unknown and is likely heterogenous [1]. This inherent complexity and mystery surrounding ASD contributes to and compounds the lack of effective treatments for core behavioral symptoms. Current knowledge suggests environmental factors are malleable and sometimes preventable contributors to ASD incidence. Understanding these factors can aid in addressing ASD prevalence and improving treatment opportunities.

Environmental factors which may be related to increased ASD incidence are diverse, commonplace, and some are unavoidable. Some are endogenous factors in the *in utero* environment–particularly those associated with inflammation. One common acute inflammatory event is maternal immune activation (MIA) during the first two trimesters–a physiological response to infection that increases the risk of persistent autistic behaviors in the offspring [2]. An epidemiological study of over 10,000 ASD cases found that a viral infection in the mother during the first trimester, and any infection (viral or bacterial) during the second trimester, was associated with an increased incidence of an ASD diagnosis in the child [3]. Similar findings have been reported in more recent studies [4–6]. MIA-induced inflammation triggers an increase of proinflammatory factors including interleukins and activates immune cells in the decidua, bathing the fetus in proinflammatory compounds and antibodies and increasing the likelihood of the child developing ASD [7]. MIA–induced ASD symptoms have been validated in multiple animal models: rodent MIA models have been developed using synthetic agents that induce an immune response such as the RNA mimic polyinosinic-polycytidylic acid (poly(I:C), evoking an antiviral-type response) and the bacterial cell wall component lipopolysaccharide (evoking an antibacterial-type response) offspring demonstrate the core symptoms of autism [8, 9].

The ability for immune/inflammatory conditions to influence ASD incidence has elicited research into commensurate strategies to prevent or treat ASD. Related to this, low-carbohydrate, high-fat ketogenic diets (KDs) have been used for many decades to treat epilepsy, including epilepsy co-morbid with ASD [10, 11] and there is evidence that they lower inflammation (for instance [12, 13]). Low dietary carbohydrate and limited protein forces nervous tissue to rely on ketone bodies (acetoacetate, β-hydroxybutyrate, acetone) produced in the liver for energy. A KD can be effective even in drug-resistant epilepsy [14], with laboratory and clinical evidence for antiepileptogenesis (e.g. [15, 16]). Studies of chronic and neurodegenerative conditions in animal models strongly suggest neuroprotective and disease-modifying effects of KDs (for example [16, 17]).

Recently there has been growing evidence for the benefits of KDs against core ASD symptoms as well as the common ASD comorbidity of epileptic seizures [18]. This includes case studies in children describing improvements in core symptoms (as well as seizures) [19, 20] and small pilot studies reporting varied responses including improvements during KD use [21–23]. More recently, studies have shown that KD treatment improves sociability and reduces self-directed repetitive behavior in animal models of ASD such as the BTBR T+ tf/J mouse strain [24], the EL mouse strain [25] and mice with genetic inactivation of the *Engrailed 2* gene [26]. Specifically for environmentally-induced ASD, KD feeding improves sociability and repetitive behavior in the gestational valproic acid model in rats [27, 28]. Here, we administered prenatal poly(I:C) injections in mice to investigate the effects of maternal inflammation and subsequent KD treatment on ASD-associated behaviors in male and female offspring.

## Materials & methods

### Animals

All procedures were performed in accordance with the NIH Guide for the Care and Use of Laboratory Animals, and approved by the Institutional Animal Care and Use Committee of Trinity College (A3869–01). Young adult female C57Bl/6 mice (original breeders from Jackson Laboratories, Bar Harbor, ME) were determined to be proven breeders after having one or two litters. Proven breeders were housed socially with same-sex cage mates. Estrous cycle was monitored daily by visual inspection of the external genitalia [29]. When a breeder was determined to be in proestrus or estrus, she was housed overnight with an adult male C57Bl/6 mouse. The following morning, the female mouse was checked for the presence of a vaginal plug, which marked embryonic day 0.5 (E0.5). Pregnant females were housed socially and not disturbed except for cage cleaning.

On days E10.5, E12.5, and E14.5, dams were weighed and injected intraperitoneally with 5 mg/kg poly(I:C) (potassium salt; P9582; Sigma, St. Louis, MO). Poly(I:C) was supplied by the manufacturer at 10% of the total weight of the salt; dosage was based on the weight of poly(I:C) itself. Control dams were not injected to minimize prenatal/gestational stress. Pups from each litter remained with the mother until postnatal day 21 or 22, when they were weaned and housed socially with same-sex littermates. Between weaning and 5 weeks of age, all offspring were fed control diet (CD; LabDiet 5001, W.F. Fisher & Son, Somerville, NJ). At 5 weeks of age, poly(I:C) littermates were allocated into control and treatment groups and kept on either CD or switched to KD (F3666; BioServ, Frenchtown, NJ; 6.6:1 fats:(carbohydrates+protein)), respectively. Fat consisted of lard, butter, and corn oil (47.5%, 20.0%, and 11.4% of total weight, respectively). Types of fatty acids were saturated (42.6% of fatty acids by weight), monounsaturated (40.4%), and polyunsaturated (17.1%). Protein was casein. All control group mice were kept on CD, and testing occurred at 8–9 weeks of age (3–4 weeks of KD treatment). This age and length of diet treatment is based on our prior work which found beneficial effects in other ASD models, and the consistent development of ketosis and moderate hypoglycemia in mice with these parameters [24, 25]. A KD group from control dams was not included as we have previously found that KD feeding does not significantly affect sociability or grooming in naïve C57Bl/6 mice [24]. Though it has been proposed that KD treatment in ASD should be combined with caloric restriction [30], all reports so far have used *ad libitum* feeding [19–28]; all diets herein were fed *ad libitum*. At the completion of all testing, mice were euthanized by isoflurane overdose.

### Sociability and communication

Sociability with conspecifics was tested using the three-chamber sociability test, and passive social communication was tested by social transmission of a food preference [31]. In the three-chamber sociability test a Plexiglas box divided into three equal chambers was used; a 6 x 6 cm door in each internal wall allowed for free movement between the chambers. Small cylindrical wire cages (diameter 10.4 cm, height 11 cm, bar intervals 1 cm) were placed in both side chambers. Test subject mice were first habituated to the testing room for 30 minutes and then habituated to the central chamber for 10 minutes with the doors closed. Testing occurred in three 10 minute phases in which the test mouse was allowed to roam freely between chambers. The test mouse was placed in the central chamber and the doors were lifted at the start of the phase. In the first phase, both wire cages were empty, allowing for a test of side bias. In phase two, a sex and diet-matched "stranger" mouse (C57Bl/6) was placed in the wire cage of one side chamber, allowing for a test of sociability. In the third phase a novel "stranger" mouse was placed in the other wire cage to allow for a test of preference for social novelty. Stranger mouse placement was counterbalanced and stranger mice were fed on the respective experimental diet for several days before testing to eliminate a possible confound of diet-related olfactory cues. All activity was video-recorded for later scoring. Sociability in this test was quantified for 1) time spent in each chamber and 2) social contact time (nose/face/forepaw contact with the cages and/or the stranger mice).

After blood testing of all groups (see below), passive social communication was assessed by social transmission of a food preference [32]. Mice were habituated to eating KD or powdered CD, as appropriate, from glass jars (Dyets, Inc., Bethlehem, PA). Flavors were cocoa (2%) or cinnamon (1%), with each serving as the trained flavor in half the trials; flavor placement in the cages was also counterbalanced. All jars were weighed before and after presentation. An isolated demonstrator mouse was fasted for 18 h, and presented with one jar of powdered flavored food (‘‘trained” flavor) until it had eaten at least 0.5 g. The demonstrator was returned to the home cage for 30 min to interact with cage-mate observer mice (training). Observer mice were fasted for 18 h, and then presented simultaneously with both the ‘‘trained” flavor (eaten by the demonstrator) and an ‘‘untrained” flavor (novel flavored food) for 2 h. In training and testing, all flavored diets were powdered CD. This maintains a consistent flavored diet scent during the training and the test. Use of the CD during training and testing is warranted even in KD-fed mice because during training, when associative learning of the food preference occurs (or not), the demonstrator mice have eaten the flavor but the observer mice (who are detecting the flavor) are not eating any food.

### Repetitive behavior

To measure self-directed repetitive behavior, self-grooming was quantified in two separate testing conditions: during the three-chamber sociability test apparatus described above, and during a separate 10 minute single chamber test. In this latter test, the mouse was habituated to the room for 30 minutes and then habituated to an experimental polypropylene cage (19 x 29 x 12.5 cm) for 10 minutes. Mouse behavior was video recorded and quantified in a 10 minute phase following habituation.

### Blood analysis

One day after the single chamber grooming test, levels of blood glucose and the ketone body β-hydroxybutyrate were measured. Mice were lightly anesthetized with isoflurane for collection of tail blood, which was analyzed with Precision Xtra meters (Abbott Laboratories, Bedford, MA).

### Data analysis

As noted, social and grooming behavior videos were scored by two verified and independent researchers: in all cases at least one was blind to treatment. Sociability preference in the three-chamber sociability test was defined as the ratio of time spent in the "social" chamber (with a mouse) to the total time spent in side chambers. Data from each group for each measure were analyzed with Grubb’s test for outliers, which were eliminated from that particular test, such that n’s may differ across tests; no mice were outliers for blood glucose or β-hydroxybutyrate. Statistical analysis was conducted using ANOVA (on ranked data when non-parametric) with Bonferroni post-hoc tests. Data are reported as mean +/- standard error; p <0.05 was considered significant. Data are available in S1 File.

## Results

### Physiology

**Fig 1:**
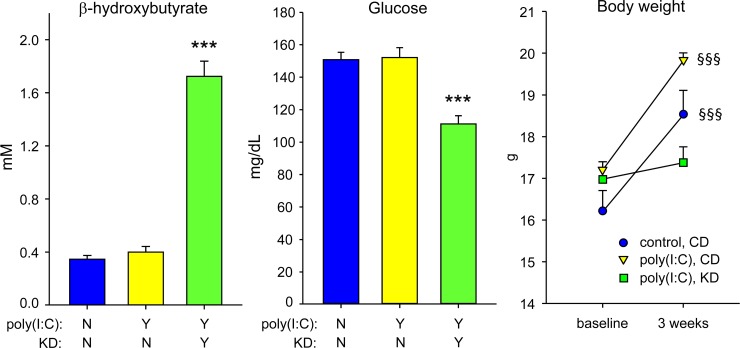
Hallmarks of KD therapy confirmed in MIA mice. *Left*: Blood β-hydroxybutyrate was elevated by KD feeding. *Middle*: Blood glucose was reduced by KD feeding. ***p<0.001 compared to CD-fed control offspring; n = 20–23. There were no sex differences in either measure (in initial analysis, sex-by-treatment interaction: F = 1.7, p = 0.19 for ketones; F = 1.8, p = 0.18 for glucose); male and female data are combined. *Right*: CD-fed female mice gained significant weight during diet treatment; KD-fed mice did not. §§§p<0.001 compared to baseline; n = 11–12. Available data from male mice showed a similar pattern (S1 File).

CD-fed MIA offspring did not differ from control offspring in their blood levels of glucose and ketones. As expected, KD-fed MIA offspring had the hallmark blood chemistry changes associated with this metabolic treatment, i.e. ketonemia (measured by increased β-hydroxybutyrate) and lowered glucose (Fig 1; ketones F = 34.6, p<0.001; glucose F = 10.4, p<0.001). There were no sex differences in levels of blood glucose or ketones. CD-fed mice gained weight during diet treatment; KD-fed mice did not change significantly (Fig 1).

### Sociability

**Fig 2:**
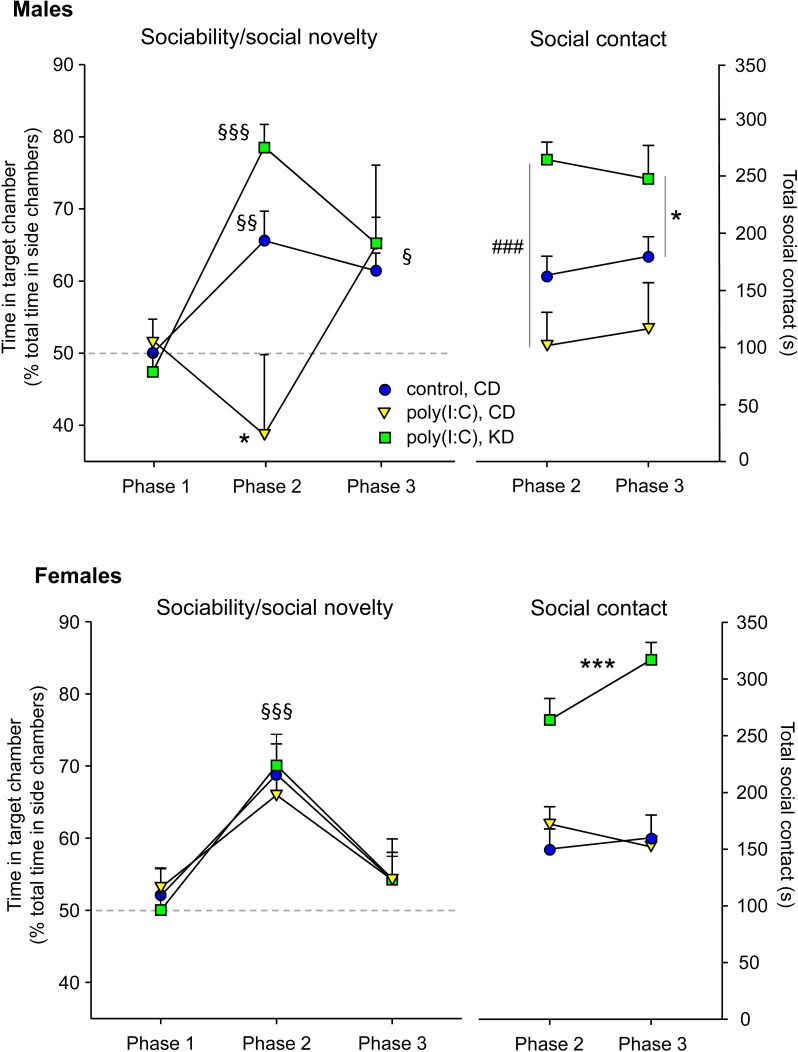
KD effects on social behavior in male and female MIA offspring. *Top panels*: male mice. *Top left*: Chamber time in the three-chamber test. CD-fed MIA offspring were not social in phase 2. This effect was reversed by KD feeding. Control offspring were social, as expected. Mice in all treatment groups showed significant preference for social novelty in phase 3. *Top right*: Social contact in the three-chamber test. Social contact time was decreased in male MIA offspring, and elevated by KD feeding to levels above control offspring. §p<0.05, §§p<0.01, §§§p<0.001 compared to phase 1 within the same treatment group. *p<0.05 compared to control mice. ###p<0.001 compared to CD-fed MIA offspring; n = 8–12. *Bottom panels*: female mice. *Bottom left*: Sociability expressed as time in chamber with a mouse was not impaired by MIA treatment and not affected by KD feeding. §§§p<0.001 overall phase 2 compared to phase 1; n = 10–12. *Bottom right*: Sociability expressed as social contact was not impaired by MIA treatment; however KD-feeding elevated social contact. ***p<0.001 overall compared to CD-fed control and MIA offspring; n = 11 all groups.

Regarding specific behavioral tests, analysis of chamber times in the three chamber sociability test revealed that CD-fed male MIA offspring were significantly asocial: in phase 2 of the test (when a mouse is present) they demonstrated no preference for the side containing the mouse (Fig 2). In contrast, KD-fed male MIA offspring were significantly social, as were control mice. With KD treatment the lack of sociability reversed completely–KD-fed male MIA offspring were not different than control male mice (treatment F = 2.6, p = 0.09; phase F = 15.4, p<0.001; interaction F = 4.5, p<0.01;). In the phase 3 test of social novelty (when a new mouse is introduced), treatment groups did not differ and all showed a significant preference for social novelty (Fig 2). Neither MIA nor KD treatment affected chamber time in female mice, which displayed normal and significantly social behavior (Fig 2; treatment F = 0.2, n.s.; phase F = 9.9, p<0.001; interaction F = 0.2, n.s.).

Sociability was assessed further by quantifying social contact time in phases of the three chamber sociability test with mice present (phases 2 and 3). Although MIA did not reduce social contact significantly in either sex (a trend in males did not reach significance), KD treatment increased social contact in both female and male MIA offspring to levels significantly above control mice, an effect not seen previously (Fig 2). There were no differences between phases 2 and 3 (Males: treatment F = 10.4, p<0.001; phase F = 0.2, n.s.; interaction F = 0.9, n.s. Females: treatment, F = 49.3, p<0.001; phase F = 3.3, n.s.; interaction F = 2.0, n.s.).

**Fig 3:**
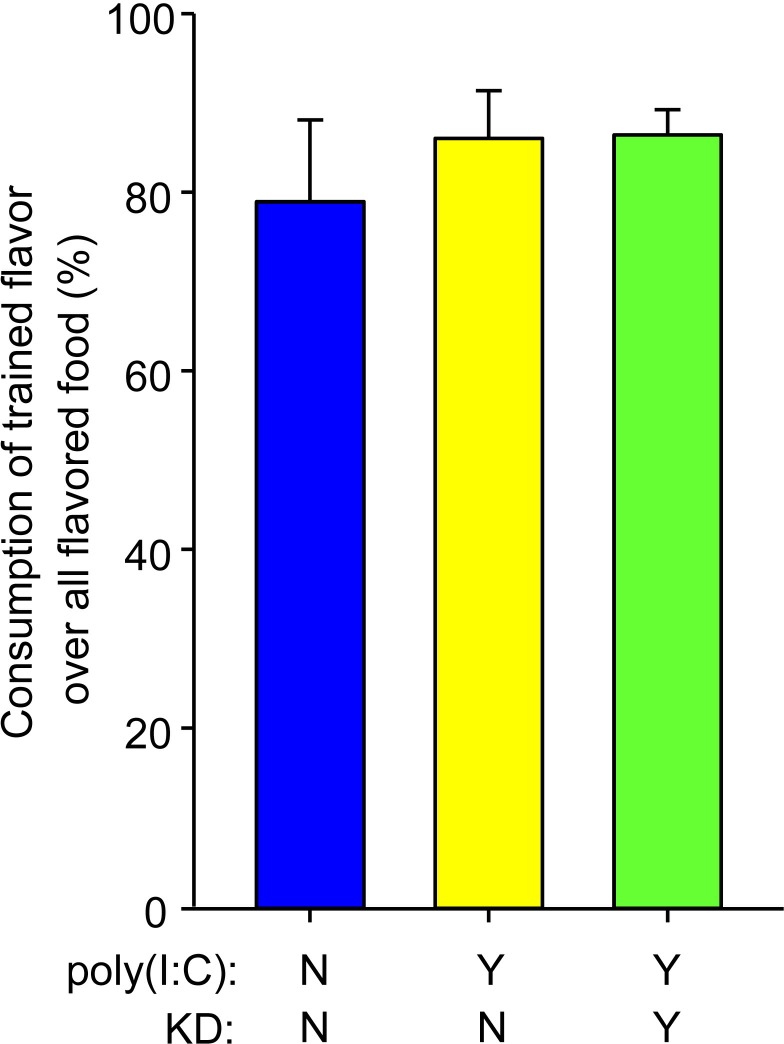
Social transmission of food preference. There was no MIA-induced impairment in this task: all treatment groups learned the social transmission of a safe food flavor; n = 11–13. There were no sex differences in this behavior (in initial analysis, sex-by-treatment interaction: F = 0.8, p = 0.45).

We also assessed sociability with a test of passive social communication, the social transmission of food preference test. However, all treatment groups performed normally and preferred the trained flavor: males and females were not different, MIA offspring were not impaired, and this behavior was not modified by KD feeding (Fig 3; H = 1.1, not significant).

### Repetitive behavior

**Fig 4:**
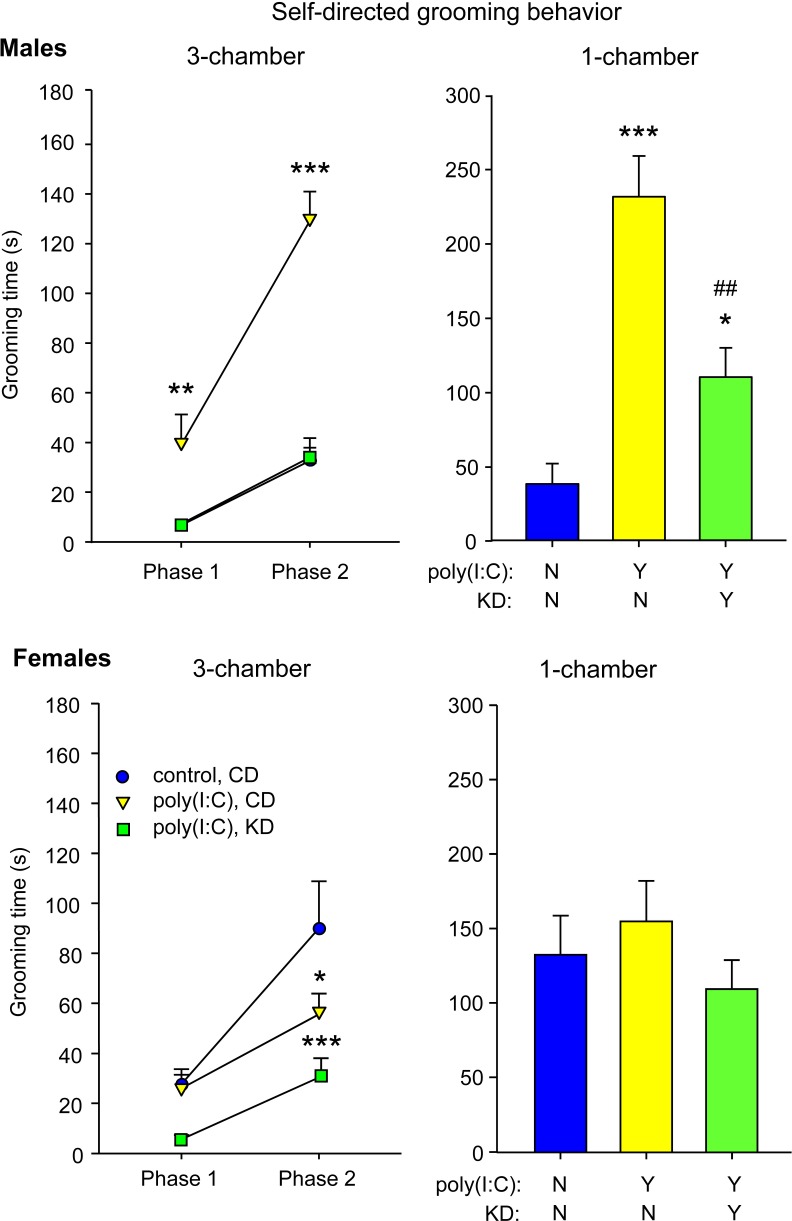
Effects of KD on self-directed repetitive grooming behavior. *Top panels*: male mice. *Top left*: Self-directed grooming behavior in the three-chamber test. Grooming was elevated by MIA treatment, and this effect was reversed completely by KD feeding. Post hoc tests were restricted to within-phase comparisons; n = 8–10. *Top right*: Self-directed behavior in the single chamber test. Grooming was elevated by MIA treatment, and this effect was partially reversed by KD feeding. **p<0.01, ***p<0.001 compared to CD-fed control offspring. ##p<0.01 compared to CD-fed MIA mice; n = 8–10. *Bottom panels*: female mice. *Bottom left*: Grooming in the three-chamber test was not elevated by MIA treatment in females; it was, however, lowered by KD feeding; n = 11–12. *Bottom right*: Grooming in the one-chamber test was not affected by MIA or KD in females. *p<0.05, ***p<0.001 compared to CD-fed control mice.

CD-fed male MIA offspring had increased self-directed repetitive behavior as measured by grooming. The increased grooming was present in the three chamber sociability test in both non-social (phase 1) and social (phase 2) situations compared to control mice (Fig 4). This elevation was reversed completely by KD feeding such that MIA offspring were no longer different from control offspring (treatment F = 48.5, p<0.001; phase F = 62.0, p<0.001; interaction F = 11.5, p<0.01). CD-fed MIA offspring also had increased self-grooming in the single chamber (non-social) test compared to control offspring (Fig 4); this effect was reversed partially by KD feeding (F = 22.6, p<0.001). MIA did not elevate grooming in female MIA offspring in either the three-chamber or single chamber tests (in fact grooming decreased in the social situation), although KD feeding reduced grooming in female MIA offspring in the three-chamber test (Fig 4; three-chamber test: treatment F = 6.0, p<0.006; phase F = 32.2; interaction F = 2.5, n.s.; single-chamber test: F = 0.8, n.s.).

## Discussion

The MIA mouse model recapitulates the clinical condition whereby ASD incidence is increased by a maternal bacterial or viral infection during pregnancy [3–6]. Furthermore, ASD-associated changes in behavior were selective to male offspring, echoing the high male prevalence (4:1 male-to-female) in humans [33]. Here we show that metabolic therapy with a KD improves and can even reverse ASD-like behaviors in the MIA mouse model.

ASD is associated with poor sociability and communication alongside restricted or repetitive behaviors, and these domains were tested in male and female MIA offspring. Previously, ASD-like behavioral effects were found in male MIA offspring, and here we also found sociability deficits specific to males [34]. However, three to four weeks of feeding a KD reduced MIA-induced social abnormalities assessed by the classic three-chamber test of sociability: KD treatment normalized sociability as assessed by time spent in a side chamber containing a mouse. In both male and female MIA offspring a KD increased time spent in direct social contact with another mouse during the three-chamber test; in females this increase resulted in higher social contact time than control animals [34]. Studies using rodent ASD models with diverse etiologies [strain (mouse: BTBR and EL), genetic (mouse: *Engrailed 2* knockout), gestational exposure (rat: valproic acid)] have also shown elevated sociability after KD feeding in three-chamber tests [24–26, 28]. In addition, a KD normalizes play behavior in juvenile rats in the gestational valproic acid model [27]. Regarding another test of sociability, the social transmission of food preference, we found no deficits in the MIA model. Sociability expressed in this test seems to vary greatly between ASD models [24, 25, 28].

KD treatment also reduced MIA-elevated self-directed repetitive behavior. In the three chamber test, elevated self-grooming was found in social and non-social phases in males and solely in the social phase in females. Elevated grooming was also found in the non-social single chamber test in males but not in females. In all cases repetitive self-grooming was reduced by KD treatment. In the BTBR mouse model we previously found elevated grooming was limited to social phases, and, similarly, was decreased and normalized by KD treatment [24]. In the EL mouse model of comorbid seizures and ASD-like behavior, self-grooming was elevated only in the non-social phase, yet this was also normalized by KD treatment [25]. Therefore in these previous studies the KD appeared to revert repetitive behavior to control levels in situations where they were elevated significantly. Reports with other models have shown similar results: in the *Engrailed 2* genetic model a KD lowered grooming in a social phase, but not a non-social phase [26]. In the gestational valproic acid model, grooming was measured in unspecified phase(s) and was reduced by KD treatment [28]. Another type of repetitive behavior, marble burying, was increased by valproic acid and normalized by KD feeding [28]. Therefore, the presence of augmented repetitive behaviors seems to depend on social condition and ASD model. However increased self-grooming is common in these models and KD is beneficial in reversing increased repetitive behaviors.

As noted, the relevance of the mouse MIA model is due to its clinically-common etiology and sex-specificity: an increased incidence of ASD is seen after a maternal infection during the first two trimesters, and the ratio of boys vs. girls diagnosed with ASD is approximately 4:1 [33]. We replicated reliable ASD behaviors in the MIA mouse model, and, like others, found that only male MIA offspring exhibit reduced sociability and consistently increased self-directed repetitive behaviors [34]. Preferential effects on males also occur with other classes of prenatal stressors [35]. The mechanism behind these sex-related differences remains uncertain, although the phenomenon is sufficiently widespread to also occur in birds [36]. In female MIA offspring sociability was normal and therefore there were no deficits for the dietary treatment to reverse. However, the same KD used in this study augmented sociability and alleviated self-directed repetitive behavior significantly in females of the EL mouse strain, a model of comorbid ASD and epilepsy [25] (KD treatment is also antiepileptogenic against the progressive seizure phenotype in this strain [37]). Therefore, the KD can exert positive effects on ASD-like behaviors in both sexes in animal models, and the interaction between sex and the behavioral effects of KDs might depend on ASD etiology. KD feeding has also been noted to improve core symptoms in girls with ASD, although the total number of reported female patients was quite small [20–22]. KD feeding also improves behavior in X chromosome-linked Rett syndrome, which may be closely related to some forms of ASD [38, 39].

Metabolic benefits of the KD regarding ASD are not solely due to increased fat. Indeed, in contrast to results obtained here with a KD, a high-fat, sufficient-carbohydrate (i.e. non-ketogenic) diet worsens ASD core behaviors in the BTBR strain [40]. Thus it is likely that the effects observed here with KD feeding are due to hallmark metabolic changes such as significant ketosis and mildly lowered systemic glucose precipitated by very high fat and restricted carbohydrate content. There are also likely a combination of short term and evolving effects of the diet. Previously we have shown that some behavioral effects required at least one week to evolve, whereas hallmark metabolic changes in blood chemistry were present within two days [41].

At this time the mechanism(s) whereby metabolic therapy with a KD translates to reduced core ASD symptoms remain hypothetical; there are many possibilities, and beneficial effects of the KD are diverse. Key mechanisms may include improved mitochondrial function [42], reduced inflammation [43], or increased adenosine [44–46]. In various in vitro and in vivo models, we have shown that a KD increases brain adenosine levels and signaling [16, 47–49]; we have also shown an inverse relationship between adenosine and symptoms of ASD [45]. While the relationship between adenosine and ASD has not been tested directly, abnormalities in purine metabolism are common in ASD [50–53], and a purinergic treatment has been shown to be effective in alleviating symptoms [54, 55]. In the gestational valproic acid model, KD treatment normalized dysfunctions in mitochondrial respiration [27]; mitochondrial dysfunction is common is ASD [56–58]. In the BTBR strain model, KD treatment normalized abnormal cerebrocortical excitation/inhibition [59], a clinically-relevant aspect of ASD [60]. KD treatment enhanced social novelty-induced neuronal activation in several brain areas of *Engrailed 2* knockout mice–but did not normalize levels of monoamine neurotransmitters [26]. More research is needed to link metabolic therapy mechanistically to symptoms of ASD.

Because of its long and successful clinical history as a metabolic therapy for epilepsy, much more work has been done on anticonvulsant mechanisms of KDs. At this time it remains unclear if anticonvulsant and anti-ASD mechanisms are identical, separate, or mixed, but ASD and epilepsy are similarly diverse and complicated: in both disorders the specific etiology likely determines key treatment mechanisms. For this reason it is possible that a broad-acting, homeostatic treatment–such as a metabolic therapy that engages multiple mechanisms–may be a particularly beneficial approach. Regardless of mechanism(s), KD improves behavior in several types of ASD models. The present study demonstrates benefits of a KD in reducing core ASD symptoms subsequent to MIA, a model with high clinical relevance. Taken together, emerging findings suggest a wide-ranging utility for metabolic therapies in improving core ASD symptoms and support further research into mechanisms and applications of these therapies for ASD patients.

## Supporting information

S1 File Data from the study. Different measures are presented on different sheets; treatments and sex are indicated for all individual subjects. Outliers have been removed, and are marked by shaded cells. NA: data not available.
(PDF)
