# Supplementary material for: Ketogenic diet improves behaviors in a maternal immune activation model of autism spectrum disorder
Source: PLoS One. 2017 Feb 6;12(2):e0171643. doi: 10.1371/journal.pone.0171643 (PMC5293204; doi:10.1371/journal.pone.0171643)
Supplement: S1 File — Different measures are presented on different sheets; treatments and sex are indicated for all individual subjects. Outliers have been removed, and are marked by shaded cells. NA: data not available. (PDF) [file pone.0171643.s001.pdf]

social contact (s)

			PHASE 2	PHASE 3
**sex**	**drug**	**diet**	**Mouse**	**Total**
F	poly(IC)	CD	163	191
F	poly(IC)	CD	103	171
F	poly(IC)	CD	203	129
F	poly(IC)	CD	194	197.23
F	poly(IC)	CD	264	198
F	poly(IC)	CD	188	121
F	poly(IC)	CD	outlier	outlier
F	poly(IC)	CD	150	135
F	poly(IC)	CD	160	131
F	poly(IC)	CD	103	163
F	poly(IC)	CD	162	113
F	poly(IC)	CD	119	172
M	poly(IC)	CD	0	2
M	poly(IC)	CD	87	100
M	poly(IC)	CD	61	7
M	poly(IC)	CD	49	39
M	poly(IC)	CD	193	217
M	poly(IC)	CD	44	126
M	poly(IC)	CD	242	337
M	poly(IC)	CD	134	103
M	poly(IC)	F3666	357	318
M	poly(IC)	F3666	272	279
M	poly(IC)	F3666	299	205
M	poly(IC)	F3666	242	186
M	poly(IC)	F3666	252	380
M	poly(IC)	F3666	295	270
M	poly(IC)	F3666	201	61
M	poly(IC)	F3666	240	169
M	poly(IC)	F3666	197	327
M	poly(IC)	F3666	284	271
F	poly(IC)	F3666	226	230
F	poly(IC)	F3666	159	396
F	poly(IC)	F3666	350	308
F	poly(IC)	F3666	232	349
F	poly(IC)	F3666	289	312
F	poly(IC)	F3666	308	259
F	poly(IC)	F3666	317	391
F	poly(IC)	F3666	195	346
F	poly(IC)	F3666	314	295
F	poly(IC)	F3666	203	306
F	poly(IC)	F3666	307	297

			PHASE 2	PHASE 3
**sex**	**drug**	**diet**	**Mouse**	**Total**
F	uninjected	CD	183	228
F	uninjected	CD	178	275
F	uninjected	CD	153	245
F	uninjected	CD	123	95
F	uninjected	CD	outlier	outlier
F	uninjected	CD	153	51
F	uninjected	CD	116	161
F	uninjected	CD	123	213
F	uninjected	CD	90	116
F	uninjected	CD	102	110
F	uninjected	CD	127	180
F	uninjected	CD	171	95
M	uninjected	CD	189	139
M	uninjected	CD	115	144
M	uninjected	CD	211	227
M	uninjected	CD	107	175
M	uninjected	CD	120	138
M	uninjected	CD	72	86
M	uninjected	CD	259	239
M	uninjected	CD	215	229
M	uninjected	CD	169	151
M	uninjected	CD	176	166
M	uninjected	CD	157	278

Times in target chamber (% of total times in side chambers)

sex	drug	diet	PHASE 1	PHASE 2	PHASE 3
F	poly(IC)	CD	39.15	71.23	48.12
F	poly(IC)	CD	37.94	42.89	75.68
F	poly(IC)	CD	56.93	85.19	37.96
F	poly(IC)	CD	58.57	63.28	55.58
F	poly(IC)	CD	56.04	85.17	44.16
F	poly(IC)	CD	60.21	70.67	39.52
F	poly(IC)	CD	48.23	81.40	44.23
F	poly(IC)	CD	47.48	73.77	68.13
F	poly(IC)	CD	45.91	54.41	61.95
F	poly(IC)	CD	59.57	71.00	52.71
F	poly(IC)	CD	66.36	57.85	47.56
F	poly(IC)	CD	61.21	34.96	75.08
M	poly(IC)	CD	64.62	0.00	100.00
M	poly(IC)	CD	53.85	37.22	79.90
M	poly(IC)	CD	55.17	22.94	62.50
M	poly(IC)	CD	36.87	13.83	0.00
M	poly(IC)	CD	48.99	73.81	59.45
M	poly(IC)	CD	49.60	14.56	100.00
M	poly(IC)	CD	58.26	61.24	61.26
M	poly(IC)	CD	46.87	85.97	55.76
F	uninjected	CD	40.89	59.61	61.30
F	uninjected	CD	60.38	75.29	56.29
F	uninjected	CD	41.13	56.26	59.89
F	uninjected	CD	outlier	outlier	outlier
F	uninjected	CD	31.28	83.55	50.05
F	uninjected	CD	74.51	80.20	18.79
F	uninjected	CD	56.63	83.00	64.10
F	uninjected	CD	48.48	46.27	55.90
F	uninjected	CD	44.62	79.36	69.80
F	uninjected	CD	54.76	47.16	77.22
F	uninjected	CD	68.49	76.61	29.12
F	uninjected	CD	50.97	69.70	
M	uninjected	CD	53.58	69.70	51.09
M	uninjected	CD	58.19	54.20	58.71
M	uninjected	CD	49.42	74.02	70.27
M	uninjected	CD	52.28	78.88	73.51
M	uninjected	CD	51.33	69.08	56.58
M	uninjected	CD	38.18	37.09	73.62
M	uninjected	CD	53.93	71.32	57.82
M	uninjected	CD	51.55	68.83	64.32
M	uninjected	CD	40.87	73.55	52.33
M	uninjected	CD	45.85	78.05	62.48
M	uninjected	CD	54.95	46.75	54.89

sex	drug	diet	PHASE 1	PHASE 2	PHASE 3
M	poly(IC)	F3666	57.84	90.06	70.72
M	poly(IC)	F3666	36.43	79.12	50.18
M	poly(IC)	F3666	53.78	80.88	62.70
M	poly(IC)	F3666	outlier	outlier	outlier
M	poly(IC)	F3666	49.76	76.61	70.52
M	poly(IC)	F3666	49.44	79.44	56.83
M	poly(IC)	F3666	46.19	62.97	83.58
M	poly(IC)	F3666	41.01	71.11	66.20
M	poly(IC)	F3666	outlier	outlier	outlier
M	poly(IC)	F3666	45.02338	88.5215794	61.2403101
F	poly(IC)	F3666	47.55	65.97	56.33
F	poly(IC)	F3666	58.52	44.44	67.74
F	poly(IC)	F3666	48.14	84.15	50.94
F	poly(IC)	F3666	48.23	55.49	71.81
F	poly(IC)	F3666	47.50	75.79	48.04
F	poly(IC)	F3666	48.81	82.52	59.96
F	poly(IC)	F3666	65.57	80.32	59.56
F	poly(IC)	F3666	35.58	52.29	42.09
F	poly(IC)	F3666	42.95	87.16	53.06
F	poly(IC)	F3666	52.00	65.44	50.49
F	poly(IC)	F3666	54.00	76.30	37.03

PHYSIOLOGY

sex	treatment	diet	baseline weight	8wk weight	glucose	ketones
F	uninjected	CD	20.6	18.8	154	0.6
F	uninjected	CD	16.5	20.1	158	0.6
F	uninjected	CD	14.7	20.3	155	0.4
F	uninjected	CD	14.3	14	161	0.3
F	uninjected	CD	17.6	19.4	155	0.3
F	uninjected	CD	16.6	18.3	184	0.3
F	uninjected	CD	14.3	15.2	109	0.4
F	uninjected	CD	15.6	19.1	126	0.4
F	uninjected	CD	17.4	20.8	127	0.4
F	uninjected	CD	15.5	19.5	134	0.3
F	uninjected	CD	15.7	19.2	166	0.4
F	uninjected	CD	15.6	17.5	157	0.6
M	uninjected	CD	14.7	23.8	160	0.2
M	uninjected	CD	17.1	21.8	140	0.2
M	uninjected	CD	17.7	21.1	156	0.2
M	uninjected	CD	13.9	18.6	143	0.2
M	uninjected	CD	17.6	22.6	135	0.2
M	uninjected	CD	19.4	24.5	130	0.2
M	uninjected	CD	19.2	23.3	120	0.4
M	uninjected	CD	21.9	25.1	162	0.3
M	uninjected	CD	23.6		204	0.4
M	uninjected	CD	20.9		151	0.2
M	uninjected	CD	19.3		175	0.5
F	poly-(I:C)	CD	17.2	19.8	167	0.3
F	poly-(I:C)	CD	16.5	19.1	127	0.6
F	poly-(I:C)	CD	17.9	20.5	142	0.2
F	poly-(I:C)	CD	16.6	20.6	108	0.6
F	poly-(I:C)	CD	17.5	20.4	144	0.4
F	poly-(I:C)	CD	16.6	19.7	121	0.5
F	poly-(I:C)	CD	16.9	19.1	154	0.6
F	poly-(I:C)	CD	17.6	20	153	0.6
F	poly-(I:C)	CD	17.4	20.8	161	0.5
F	poly-(I:C)	CD	16.1	18.8	152	0.6
F	poly-(I:C)	CD	17.6	19.2	131	0.7
F	poly-(I:C)	CD	18.5	19.8	162	0.6
M	poly-(I:C)	CD			114	0.2
M	poly-(I:C)	CD			192	0.2
M	poly-(I:C)	CD			148	0.3
M	poly-(I:C)	CD			199	0.2
M	poly-(I:C)	CD			128	0.3
M	poly-(I:C)	CD			172	0.2
M	poly-(I:C)	CD			155	0.3
M	poly-(I:C)	CD			207	0.1

sex	treatment	diet	baseline weight	8wk weight	glucose	ketones
F	poly-(I:C)	F3666	17.7	17.8	104	2.5
F	poly-(I:C)	F3666	17.5	17	93	2.3
F	poly-(I:C)	F3666	16.4	16.3	111	2.4
F	poly-(I:C)	F3666	16.8	17.1	100	2.8
F	poly-(I:C)	F3666	15.8	16.2	88	2.3
F	poly-(I:C)	F3666	15.4	15.5	112	2.4
F	poly-(I:C)	F3666	17.7	19.9	134	1.2
F	poly-(I:C)	F3666	16.8	17.7	132	1.4
F	poly-(I:C)	F3666	17.7	18.3	136	1.4
F	poly-(I:C)	F3666	18.4	18.7	136	1.3
F	poly-(I:C)	F3666	16.6	16.6	112	1.4
M	poly-(I:C)	F3666	20.1	19.5	129	1.4
M	poly-(I:C)	F3666	20.1	19.8	131	1.3
M	poly-(I:C)	F3666	19.1	17.8	87	1.4
M	poly-(I:C)	F3666	19.9	19.5	123	1.2
M	poly-(I:C)	F3666	21.2	18.8	115	1.5
M	poly-(I:C)	F3666	21.4	22.5	94	1.1
M	poly-(I:C)	F3666	16.2	16.2	87	1.7
M	poly-(I:C)	F3666	19.8	17.1	49	2.1
M	poly-(I:C)	F3666	20.3	20.8	105	1.4
M	poly-(I:C)	F3666	21.6	19.1	153	1.7

1-chamber test grooming (s)

sex	injection	diet	
F	poly(IC)	CD	2
F	poly(IC)	CD	260.5
F	poly(IC)	CD	330.5
F	poly(IC)	CD	124.5
F	poly(IC)	CD	159
F	poly(IC)	CD	134.5
F	poly(IC)	CD	42
F	poly(IC)	CD	159
F	poly(IC)	CD	64
F	poly(IC)	CD	202
F	poly(IC)	CD	150
F	poly(IC)	CD	239
M	poly(IC)	CD	217.5
M	poly(IC)	CD	317.5
M	poly(IC)	CD	315
M	poly(IC)	CD	160.5
M	poly(IC)	CD	129.25
M	poly(IC)	CD	147.5
M	poly(IC)	CD	282
M	poly(IC)	CD	280
F	poly(IC)	F3666	43.5
F	poly(IC)	F3666	160
F	poly(IC)	F3666	84.5
F	poly(IC)	F3666	88.5
F	poly(IC)	F3666	136.5
F	poly(IC)	F3666	166.5
F	poly(IC)	F3666	47.5
F	poly(IC)	F3666	46
F	poly(IC)	F3666	141
F	poly(IC)	F3666	243
F	poly(IC)	F3666	53.5
M	poly(IC)	F3666	97.5
M	poly(IC)	F3666	192.5
M	poly(IC)	F3666	55
M	poly(IC)	F3666	70
M	poly(IC)	F3666	38
M	poly(IC)	F3666	113
M	poly(IC)	F3666	187
M	poly(IC)	F3666	209
M	poly(IC)	F3666	81
M	poly(IC)	F3666	113

sex	injection	diet	
F	uninjected	CD	179
F	uninjected	CD	42
F	uninjected	CD	270
F	uninjected	CD	25.5
F	uninjected	CD	136.5
F	uninjected	CD	89
F	uninjected	CD	317.5
F	uninjected	CD	126
F	uninjected	CD	160
F	uninjected	CD	44
F	uninjected	CD	148
F	uninjected	CD	60.5
M	uninjected	CD	59
M	uninjected	CD	24.5
M	uninjected	CD	4
M	uninjected	CD	26
M	uninjected	CD	8
M	uninjected	CD	111.5
M	uninjected	CD	12.5
M	uninjected	CD	14.5
M	uninjected	CD	4
M	uninjected	CD	117

3-chamber grooming (s)

se	inject	diet	PHASE 1	PHASE 2
F	poly(IC)	CD	52	78.5
F	poly(IC)	CD	3	67.5
F	poly(IC)	CD	45.5	71.5
F	poly(IC)	CD	3.5	42
F	poly(IC)	CD	2.5	10
F	poly(IC)	CD	54	34.5
F	poly(IC)	CD	21.5	40
F	poly(IC)	CD	29.5	58.5
F	poly(IC)	CD	41.5	97.5
F	poly(IC)	CD	16.5	101.5
F	poly(IC)	CD	13	22.5
F	poly(IC)	CD	29.5	44
M	poly(IC)	CD	2.5	88
M	poly(IC)	CD	102.5	139
M	poly(IC)	CD	25.5	100
M	poly(IC)	CD	69	133
M	poly(IC)	CD	60.5	91
M	poly(IC)	CD	24	170
M	poly(IC)	CD	11.5	140
M	poly(IC)	CD	17	172.5
M	poly(IC)	F3666	3.5	27.5
M	poly(IC)	F3666	11	22
M	poly(IC)	F3666	6	44
M	poly(IC)	F3666	4	61.5
M	poly(IC)	F3666	5	13
M	poly(IC)	F3666	7	18.5
M	poly(IC)	F3666	8.5	85.5
M	poly(IC)	F3666	8	2
M	poly(IC)	F3666	7	34.5
M	poly(IC)	F3666	13	31.5
F	poly(IC)	F3666	17	17.5
F	poly(IC)	F3666	3	1.5
F	poly(IC)	F3666	2	13
F	poly(IC)	F3666	11.5	14
F	poly(IC)	F3666	0.5	39
F	poly(IC)	F3666	13.5	56
F	poly(IC)	F3666	0.5	58.5
F	poly(IC)	F3666	0	25.5
F	poly(IC)	F3666	5.5	97.5
F	poly(IC)	F3666	8.5	34.5
F	poly(IC)	F3666	14	31

sex	inject	diet	PHASE 1	PHASE 2
F	uninject	CD	35.5	96
F	uninject	CD	14.5	88
F	uninject	CD	11	1
F	uninject	CD	50.5	0
F	uninject	CD	4.5	37.5
F	uninject	CD	32.5	55.5
F	uninject	CD	0	85.5
F	uninject	CD	65.5	212
F	uninject	CD	10.5	117
F	uninject	CD	53.5	138
F	uninject	CD	25	178
F	uninject	CD	29.5	73
M	uninject	CD	1	40
M	uninject	CD	0.5	34.5
M	uninject	CD	0	55
M	uninject	CD	0	47
M	uninject	CD	10.5	53
M	uninject	CD	23	35.5
M	uninject	CD	outlier	outlier
M	uninject	CD	8.5	19
M	uninject	CD	14.5	20
M	uninject	CD	4	5.5
M	uninject	CD	5	16.5

STFP

maternal treatment	diet	2-jar test: trained food eaten (% of total food eaten)
poly(I:C)	F3666	82.98
poly(I:C)	F3666	93.33
poly(I:C)	F3666	97.73
poly(I:C)	F3666	93.44
poly(I:C)	F3666	74.63
poly(I:C)	F3666	69.49
poly(I:C)	F3666	75.86
poly(I:C)	F3666	91.30
poly(I:C)	F3666	92.68
poly(I:C)	F3666	90.16
poly(I:C)	F3666	92.41
poly(I:C)	control	97.89
poly(I:C)	control	98.32
poly(I:C)	control	89.80
poly(I:C)	control	40.22
poly(I:C)	control	90.20
poly(I:C)	control	98.85
poly(I:C)	control	51.16
poly(I:C)	control	100.00
poly(I:C)	control	84.06
poly(I:C)	control	93.26
poly(I:C)	control	100.00
poly(I:C)	control	100.00
poly(I:C)	control	78.41
uninjected	control	82.61
uninjected	control	93.33
uninjected	control	88.61
uninjected	control	87.61
uninjected	control	91.22
uninjected	control	100.00
uninjected	control	27.69
uninjected	control	100.00
uninjected	control	97.70
uninjected	control	10.39
uninjected	control	92.36
uninjected	control	93.46
